# Beyond Traditional Synthesis: Electrochemical Approaches to Amine Oxidation for Nitriles and Imines

**DOI:** 10.1021/acsorginorgau.4c00025

**Published:** 2024-06-21

**Authors:** Zhining Xu, Ervin Kovács

**Affiliations:** † Institute of Materials and Environmental Chemistry, HUN-REN Research Centre for Natural Sciences, Magyar tudósok körútja 2, H-1117 Budapest, Hungary; ‡ Hevesy György PhD School of Chemistry, Eötvös Loránd University, Pázmány Péter sétány 1/A, H-1117 Budapest, Hungary

**Keywords:** Amine oxidation, Aminoxyl, Electrocatalysis, Electrochemistry, Electrosynthesis, Imines, Modified electrode, Nitriles, Oxidation, Sustainable Chemistry

## Abstract

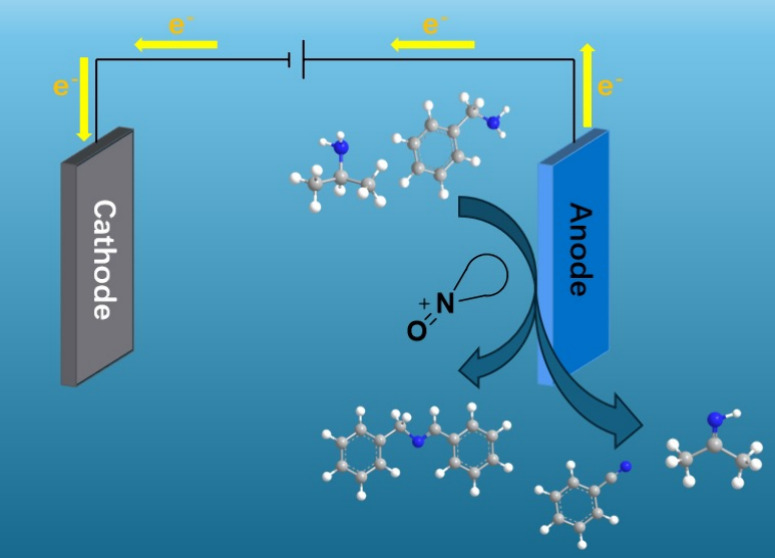

The electrochemical oxidation of amines to nitriles and imines represents a critical frontier in organic electrochemistry, offering a sustainable pathway to these valuable compounds. Nitriles and amines are pivotal in various industrial applications, including pharmaceuticals, agrochemicals, and materials science. This review encapsulates the recent advancements in the electrooxidation process, emphasizing mechanistic understanding, electrode material innovations, optimization of reaction conditions, and exploration of solvent and electrolyte systems. Additionally, the review addresses the operational parameters that significantly affect the electrooxidation process, such as current density, temperature, and electrode surface, offering insights into their optimization for enhanced performance. By providing a comprehensive view of the current state and prospects of amine electrooxidation to nitriles and imines, this review aims to inspire further development, innovation, and research in this promising area of green chemistry.

## 1 Introduction

Nitriles and imines are two important organic compounds with extensive applications in many industrial and scientific fields.^1,2^ Nitrile compounds are commonly used in manufacturing polyamide fibers (nylon), synthetic drugs, and pharmaceutical intermediates and in the production of coatings and paints. As one of the raw materials for polymers, nitrile compounds have high significance in producing textiles, plastic products, and engineering materials. In addition, nitrile compounds are widely used in organic synthesis reactions as intermediates for preparing amino acids, amides, ethers, and other compounds.^3,4^ Imine compounds also play an important role in the pharmaceutical industry as intermediates and raw materials for drug synthesis and are used to prepare bioactive molecules. Meanwhile, imines are also widely used in the pesticide industry for synthesizing insecticides, herbicides, and fungicides. In addition, imines are also important intermediates in organic synthesis, used to synthesize various organic compounds such as ketones, alcohols, and amines.^1^ Overall, nitriles and imines have a wide range of applications in fields such as medicine production, pesticides, and organic synthetic processes and are indispensable and important components in the chemical industry and scientific research.

**Scheme 1:**
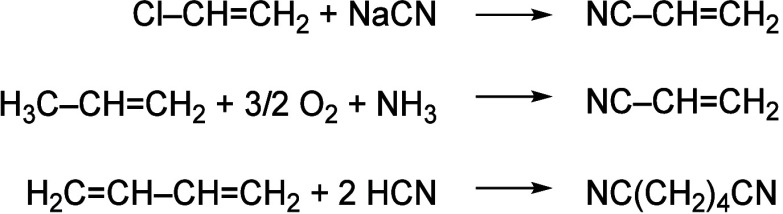
Traditional Methods for Nitrile Synthesis

Nitriles are mainly synthesized by ammoxidation, and cyanation of olefins using toxic and harsh acidic dehydration reagents or substitution of alkyl halides (Scheme 1), causing significant environmental pollution.^5−8,72^

**Scheme 2:**
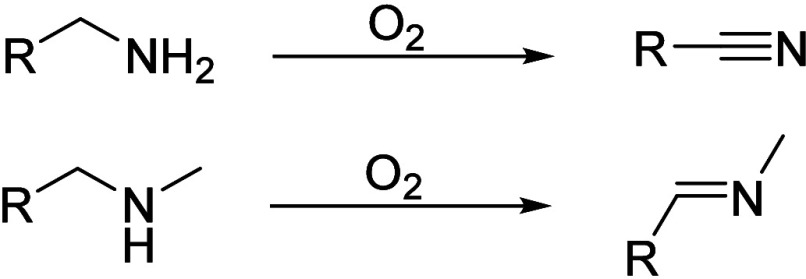
Primary and Secondary Amines Are Oxidized to Nitriles (top) and Imines (bottom)

Nitriles can also be obtained from primary amines, while the oxidation of secondary amines can yield imines as a product (Scheme 2).^9,10^

The first nitrile compound, benzonitrile, was synthesized in 1832 by Wohler and Liebigg; afterward, in 1834, Pelouze successfully prepared acetonitrile.^2^ In the 1930s, nitrile was synthesized through cyanide, aryl halides, and organometallic compounds. Since the 1960s, nitrile has mainly been obtained through ammonia oxidation, and in recent years, various catalysts have been continuously developed for ammonia oxidation reactions to synthesize nitrile.^11^ Afterward, researchers developed various electrochemical methods. In addition to using *N*-oxoammonium salts as mediators to synthesize nitrile compounds,^12,13^ molecular iodine is also an appropriate oxidizing agent for this purpose.^14,15^

Substrates containing amines are difficult to oxidize relative to alcohols, as various products may be produced depending on the choice of oxidants and reaction conditions.^16^ The oxidation of an amine to an imine requires a dehydrogenation reaction. Oxidizing primary amines to nitriles is more challenging. The multifunctionality of nitrile functional groups and their presence in pharmacologically active compounds have sparked interest in developing inexpensive, efficient, and environmentally friendly methods to obtain nitriles using Dess–Martin periodinane.^16^

Imine compounds are as important as amines and play irreplaceable roles in agriculture, medicine, and chemical biology.^18^ The traditional reaction of amino oxidation to imine requires an equal molar amount of oxidant (e.g., Dess–Martin periodinane, Swern oxidant, metal catalysts,^19,20^ Lewis acid catalysts,^21^ and TEMPO^22,23^) or higher reaction temperature;^24,25^ furthermore, due to the low atom efficiency, these processes will generate a significant quantity of potentially toxic byproducts. Therefore, efficient, economical, and green control of oxidation conditions to oxidize amines into the desired target compound has become a goal pursued by some researchers.

In conclusion, the traditional method of producing nitrile and imine compounds relies on chemical catalysts under high temperature and pressure conditions or special, strong oxidizing agents. Due to undesirable conditions such as high temperatures, using toxic reagents, and expensive catalysts associated with traditional synthesis methods, electrochemical synthesis has attracted chemists’ attention. Organic electrosynthetic methods usually provide green, low-consumption, and efficient ways. Electrochemical synthesis has emerged as an environmentally friendly approach as it avoids using stoichiometric amounts of wasteful oxidizing or reducing reagents because electrons act as reactants. Electrocatalytic methods can also improve the selectivity and efficiency of responses, making them a promising modern production method.^26−33^

Pioneering research in organic electrochemistry was carried out in 1834 by Michael Faraday on the electrolysis of acetic acid.^34^ A decade later, in 1847, Kolbe’s decarboxylative dimerization was published^34^ and became the foundation of electrochemical transformations. In the middle of the 20th century, in 1949, the Simons fluorination method was reported.^35^ Industrially relevant electrochemical hydrodimerization of acetonitrile into adiponitrile was developed by Monsanto and published in 1980.^36^ These industrially relevant processes indicate the potential and the revival of organic electrochemistry.^26,37^ In recent years, many notable electrochemical transformations, including the development of the modification of nitrogen-containing organic compounds, have been published recently for the preparation of heterocycles^38,39^ and amides.^40^ In several cases, electrochemical reactions are key steps for preparing and modifying alkaloid-type compounds and total synthesis of natural products.^34,41^

**Scheme 3:**
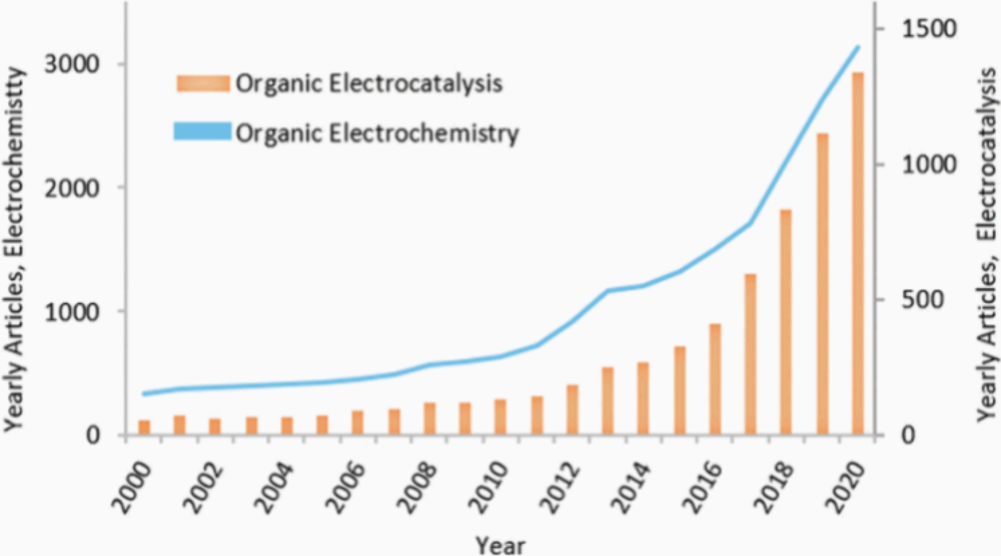
Trend of Articles on Organic Electrochemical Reactions Published in the Last 20 Years Reprinted with permission from ref (47). Copyright 2021 Royal Society of Chemistry.

Electrochemical synthesis (without additives) is an ideal sustainable method that can handle challenging syntheses with minimal waste. The scope of electrocatalysis (with additives) in chemical literature is broad, including electrocatalytic systems for energy-related applications such as water splitting^42,43^ and CO_2_ reduction,^44^ as well as synthetic chemistry (Scheme 3). Developing efficient and sustainable strategies in organic synthesis has led to a surge in interest in electrochemical methods in the organic chemistry community.^45−47^

This review mainly focuses on recent advancements in electrosynthesis and electrocatalytic oxidation reactions of amine compounds to nitriles or imines. The goal is to summarize and consolidate knowledge in this area, providing the necessary expertise for future synthetic research in related fields.

In the case of electrochemical reactions, several relevant parameters can be varied. Different types of electrodes are applied as working electrode (WE), counter electrode (CE), and reference electrode (RE), which can be modified to achieve the best yields and the highest Faradaic efficiency (FE).

## 2 Electrochemical Oxidation to Nitriles or Imines

### 2.1 Electrochemical Synthesis of Nitriles from Amines

As crucial chemical raw materials and substrates, nitriles have inspired numerous electrochemical synthesis methods utilizing primary amines and oximes as substrates.

#### 2.1.1 Application of Unmodified Electrodes in Oxidation

**Scheme 4:**
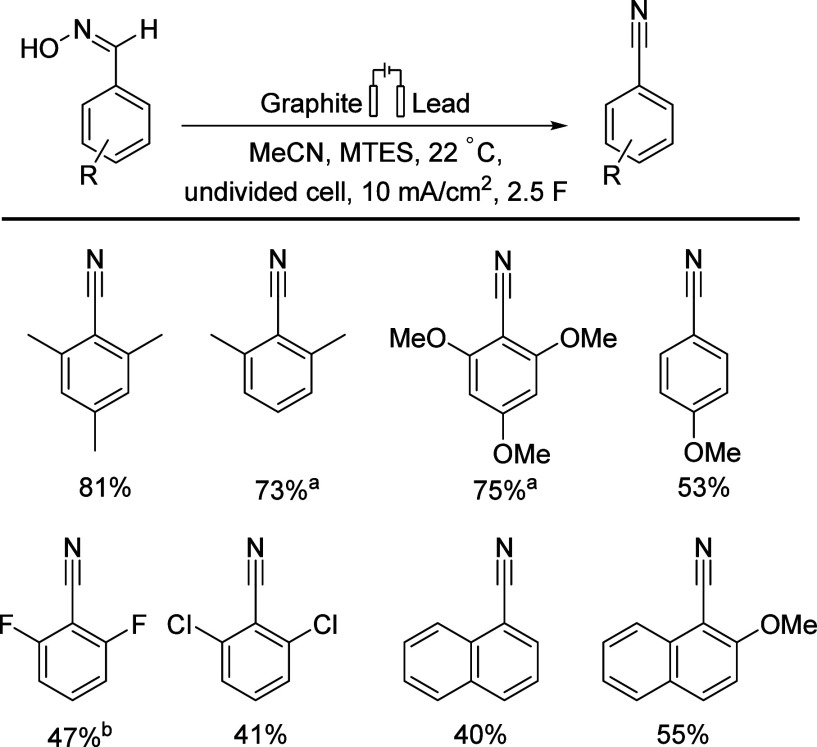
Oxime Transformation Reactions with Isolated Yields Using Lead and Graphite Electrodes Full conversion after 2.6 F. Full conversion after 2.1 F. MTES = methyltriethylammonium methylsulfate.

In 2015, Waldvogel’s group chose mesitylaldoxime as substrate, and methyltriethylammonium methylsulfate was employed as an electrolyte dissolved in acetonitrile, graphite and glassy carbon were used as anodes, and glassy carbon, lead, stainless steel, nickel, platinum, and boron-doped diamond were applied as cathodes (Scheme 4).^48^

Lead is the most effective WE, compared to other commonly used electrodes at room temperature. In summary, this work developed a direct, halogen-free, room-temperature synthesis of nitrile from oxime using inexpensive and readily available electrode materials, such as lead and graphite, in an undivided cell with an isolated yield of up to 81% for aromatic nitriles. This study achieved significant results; however, the yields from substrates with electron-withdrawing groups were lower compared to electron-donating ones. In addition, the preparation of the desired oximes usually requires additional synthetic steps.

**Scheme 5:**
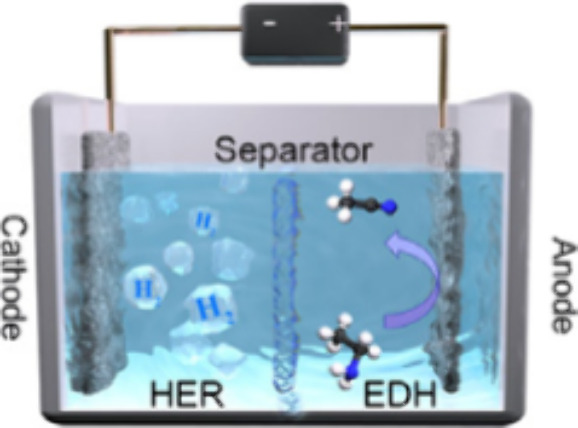
Membrane-less Cell for Electrochemical Oxidation of Ethylamine to Acetonitrile HER: hydrogen evolution reaction. EDH: ethylamine dehydrogenation. Reprinted from ref (50). Copyright 2023, with permission from Elsevier.

Platinum is a well-known and commonly used catalyst, which has also been applied in electrochemical organic reactions in recent years.^49^ Using platinum electrodes, Peng’s research group evaluated different parameters including substrate concentration (from 0 to 6 M, 2 M is the fastest), pH of electrolyte (concentrate of KOH from 0 to 3 M, 1 M is the best one), and temperature (from 276 to 303 K, and 303 K is the fastest one) for electrochemical ethylamine dehydrogenation (EDH) reaction. Two types of electrochemical cells were investigated: one with an anion exchange membrane and one without it. The membrane-less cell exhibited superior performance, achieving 100% selectivity in producing acetonitrile and 96% Faradaic efficiency (Scheme 5).^50^

#### 2.1.2 Application of Alloy and Modified Electrodes in Amine Oxidation

In recent years, significant progress has been made in the design, synthesis, and development of various high-efficiency nickel-based electrodes, including oxides/hydroxides, chalcogenides, phosphorus oxides, alloys, etc.^51−55^ Most nickel-based catalysts are converted into layered hydroxide (LOH) phases with Ni^III^O_*x*_H_*y*_ structure and electron similarity under electrochemical alkaline conditions.^56,57^ Similar to other transition metal electrocatalysts, the activity of Ni-based catalysts largely depends on the defects, surface area, morphology, crystal structure (e.g., Ni–Ni distance) of the precatalyst, amount of shared [NiO_6_] units at the edges/corners of the transformed Ni^III^O_*x*_H_*y*_ phase, and size of crystal domains.^58,59^

**Scheme 6:**
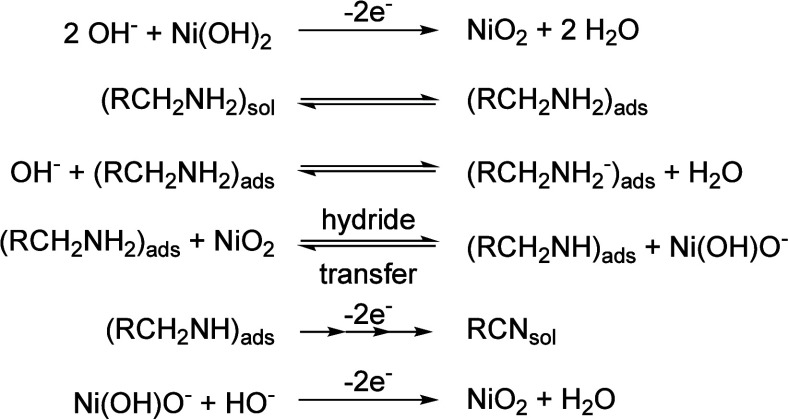
Proposed Hydride Transfer Mechanism for the Potential-Dependent Oxidation of Primary Amines to Nitriles on a NiOOH Electrosynthesis

In 2022, Choi’s group reported a kind of NiOOH electrode prepared by depositing the thin Ni(OH)_2_ film onto a flat fluorine-doped tin oxide (FTO) substrate. They investigated several parameters, including the structure and concentration of the substrate, pH, and oxidation potential, using propylamine and benzylamine as model compounds. The experiments were conducted in an undivided cell with the prepared electrode as the working electrode and Pt mesh as the counter electrode. They indicated that the oxidation mechanism of amines is very similar to alcohol oxidation (Scheme 6). In addition, a hydride transfer mechanism with Ni^4+^ as the active site was established through electrochemical rate deconvolution and density functional theory (DFT) calculations.^60^

**Scheme 7:**
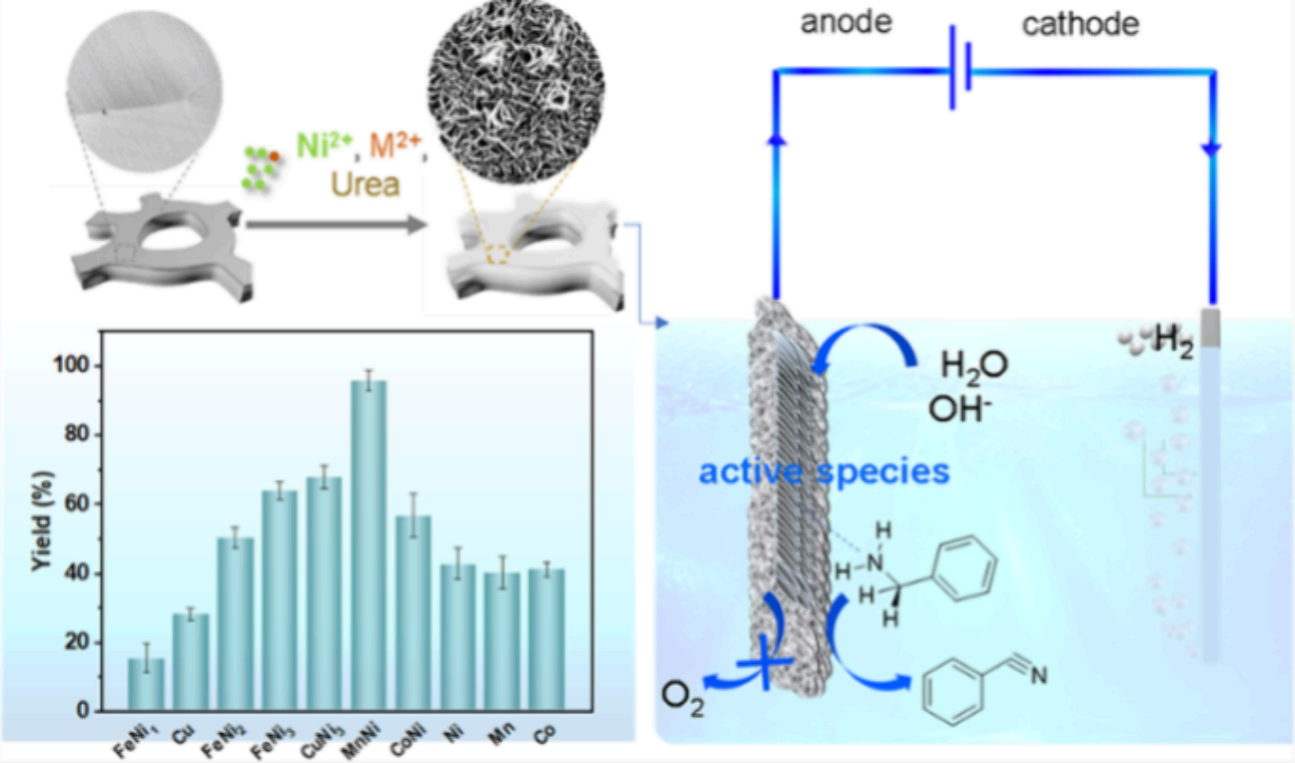
Electrocatalytic Oxidation of Amines to Nitriles Assisted by Water Oxidation on Metal-Doped α-Ni(OH)_2_ Reprinted with permission from ref (61).Copyright 2022 American Chemical Society.

In the same year, Ding’s group explored a range of transition-metal-doped α-Ni(OH)_2_, and applied them to electrodes to study the electrooxidation process of benzylamine to benzonitrile (Scheme 7). The doped transition metals included Mn, Fe, Co, and Cu; according to the results, Mn doping material is better than others with >99% conversion and over 96% FE under room temperature with a Pt sheet as a cathode in an undivided cell.^61^ Industrially relevant OER catalysts, oxyhydroxides of cobalt (CoO_*x*_), nickel–iron (NiFeO_*x*_), and nickel (NiO_*x*_), as anodes selectively catalyzed the oxidation of butylamine to butyronitrile at pH = 12. The highest activities were observed for NiO_*x*_ thin-film electrodes in the presence of butylammonium sulfate.^62^

**Scheme 8:**
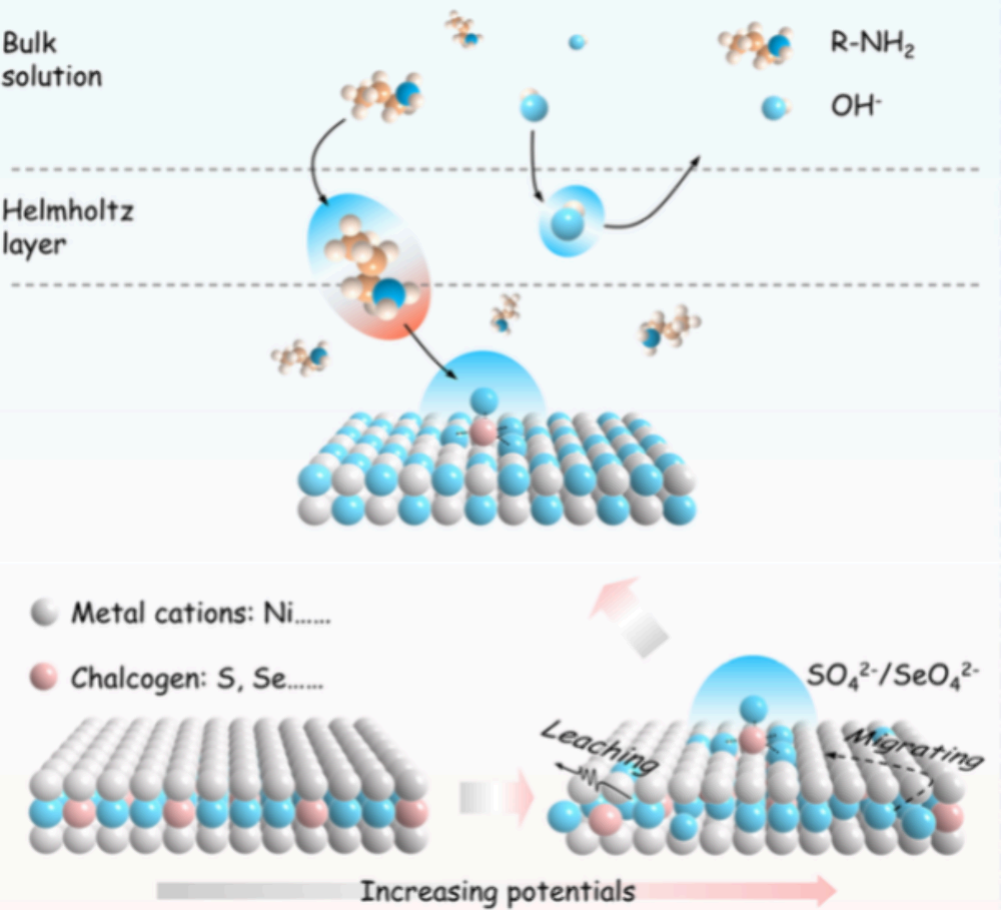
Design of I Situ Chalcogen Leaching for Manipulating the Reactant Interface toward Amine Electrooxidation Reprinted with permission from ref (64). Copyright 2022 American Chemical Society.

Due to the reactant amines attracting the bulky electrolytes via the dipole–dipole interactions, it is difficult for amine molecules to migrate to the electrode surface.^63^ In this way, Zhai’s group synthesized chalcogen-doped Ni(OH)_2_ nanosheet arrays, and α-Ni(OH)_2_ was selected as the model material, with a high performance of over 90% Faradaic efficiency and 99.5% selectivity at 1.317 V for the formation of propionitrile from propylamine. The electrodes were prepared and used as the working electrode. A carbon rod and Ag/AgCl electrode were used as the counter and the reference electrodes, respectively (Scheme 8).^64^

An unfavorable Tafel slope affects the transformed Ni^III^O_*x*_H_*y*_ phase as it has poor electronic conductivity. Due to this fact, this system requires an expanding overpotential to generate high current density.^65^ Therefore, searching for further highly active and robust nickel-based catalysts that can electrocatalyze and produce an active Ni^III^O_*x*_H_*y*_ phase under alkaline conditions is crucial.

In 2021, Huang’s group synthesized Ni_3_N nanoparticles (NPs) and Ni–Ni_3_N through a precise nitridation process. According to both the experimental and density functional theory (DFT) results, with the Ni–N bonds generated, the d-bond of Ni shifts upward, thereby improving the Ni site’s electrophilic properties and promoting the benzylamine adsorption and dehydrogenation process.

**Scheme 9:**
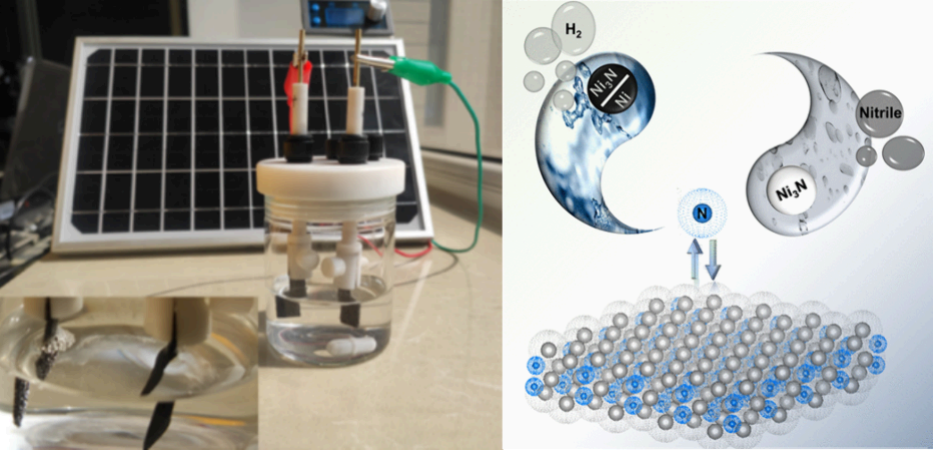
Commercial Polysilicon Solar Panels with the Ni_3_N Catalyst As the Anode (left) and Ni–Ni_3_N Heterostructures As the Cathode (right) Adapted with permission from ref (66). Copyright 2021 American Chemical Society.

Ni–Ni_3_N was used as a counter electrode (Scheme 9). In this way, Ni–Ni_3_N displays strong ability in the benzylamine oxidation reaction with ∼95% selectivity under a large current (∼250 mA).^66^

**Scheme 10:**
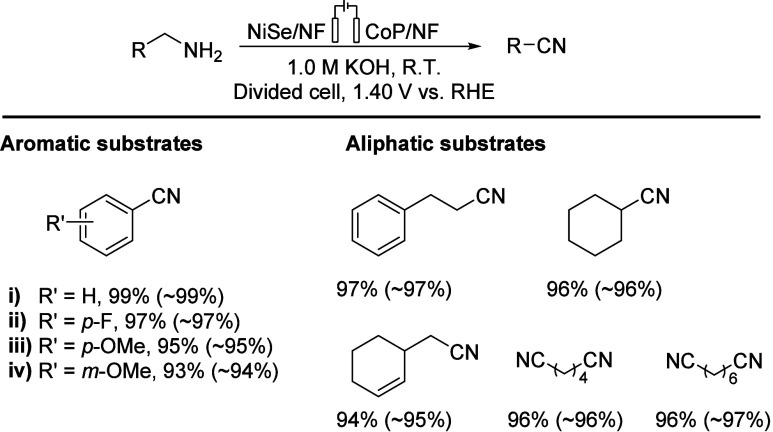
Electrosynthetic Conversion of Primary Amines into Nitriles Integrated with H_2_ Production in Water NF: nickel foam. RHE: reversible hydrogen electrode.

In 2018, Zhang’s group demonstrated primary amine oxidation reactions through a NiSe nanorod electrode in water with CoP as the cathode, and it can generate corresponding nitriles with high yields (over 93%) at room temperature (Scheme 10). This conversion demonstrates excellent substrate compatibility; alkenes, ethers, and fluorine functions can be well-preserved substitutions in certain chemical environments.^67^

**Scheme 11:**
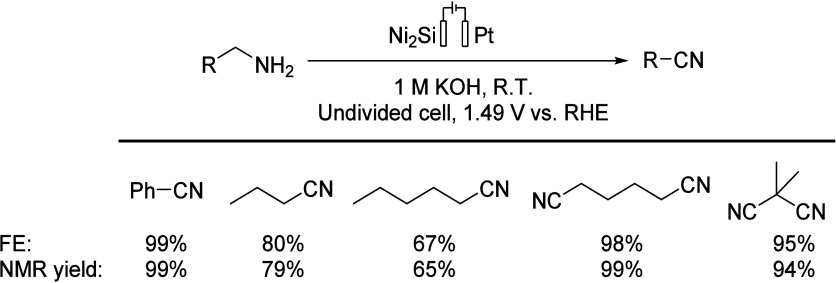
Different Substrate Scope (50 mM) for the Production of Nitriles from Their Corresponding Primary Amines with Ni_2_Si/NF Anode in 1 M KOH NPs: nanoparticles. NF: nickel foam. RHE: reversible hydrogen electrode. FE: Faradaic efficiency.

In 2022, Menezes’s group proposed Ni_2_Si NPs as electro(pre)catalysts; in an alkaline environment, the intermetallic Ni_2_Si converted to a Ni^III^O_*x*_H_*y*_ phase. The Ni-based catalyst was doped on Ni foam and fluorine-doped tin oxide, and a Pt wire was the counter electrode in an undivided cell. Compared to Ni NPs, Ni_2_Si NPs showed better electrochemical performance (after 40 min of reaction, 40% conversion with Ni NPs and 60% conversion with Ni_2_Si NPs). This research may help to develop some new types of intermetallic materials (Scheme 11).^68^

**Scheme 12:**
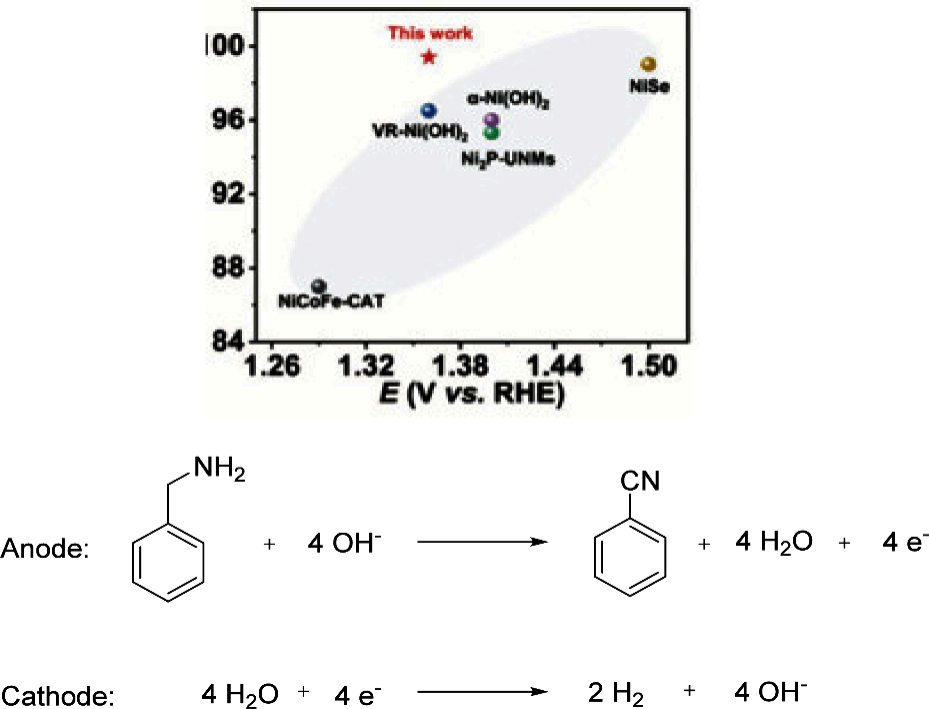
Mild Nitrile Evolution Reaction and Reduced Energy Consumption for Hydrogen Production RHE: reversible hydrogen electrode. Reprinted with permission from ref (69). Copyright 2023 John Wiley and Sons.

In 2023, Zhao’s group^69^ demonstrated a kind of self-supporting Fe–Ni_3_S_2_ electrocatalyst with 100% selective nitrile evolution reaction at a low potential; compared to the reported literature, the FE is the highest one (Scheme 12).

DFT results showed that the active site NiOOH absorbs benzylamine via the O–H···N hydrogen bond. Doping with Fe can reduce the potential of the rate-determining step from 0.59 to 0.14 eV. This selective oxidation reaction in an H-type cell employed Pt and Hg/HgO as CE and RE, respectively.^69^

In 2024, nitrogen-doped carbon-supported NiO nanoparticles with positive electronic nickel active sites were synthesized and applied successfully in electrochemical amine oxidation. High selectivity (∼99%) and complete conversion were achieved, and the onset potential of the whole reaction was as low as 1.32 V vs RHE. Detailed theoretical calculations proved that the carbon substrate enhanced the interaction between the N 2p orbital of amines and the Ni 3d orbital, which promotes the subsequent stepwise dehydrogenation to nitriles.^70^

**Scheme 13:**
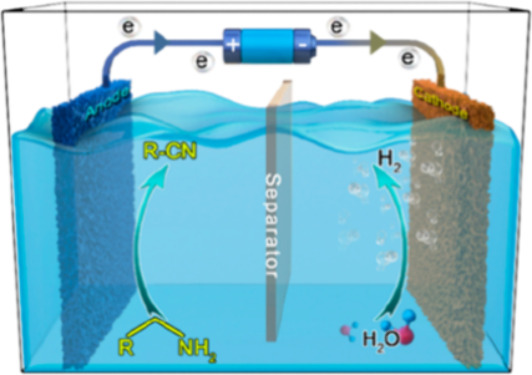
Schematic illustration Showing the Concurrent Electrolysis of Organic Amine Oxidation and the Hydrogen Evolution Reaction Reprinted with permission from ref (71). Copyright 2022 American Chemical Society.

Cobalt-related materials also have excellent reactivity toward the oxidation of amines to nitriles. In 2022, Guo’s group reported that CoSe_2_/Ni-SV SBs (subnanometer belts of CoSe_2_ with selenium vacancies and nickel substitutions) demonstrated an ultralow onset potential of 1.3 V, achieving a FE of approximately 98.5% for butyronitrile. This exceptional performance was attributed to the Se vacancies, which served as Lewis acid sites, enhancing the absorption of nitrogen atoms (Scheme 13). At the same time, the Ni substitutions can optimize the sequence of dehydrogenation steps to improve the dehydrogenation thermodynamics. In this research a graphite rod and Hg/HgO were applied as CE and RE.^71^

**Scheme 14:**
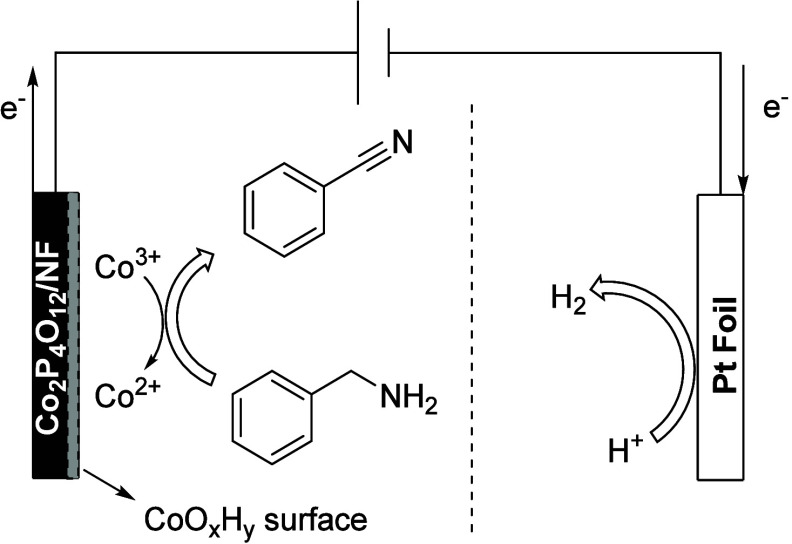
Mechanistic Illustration of Nucleophile Dehydrogenation to Reduce Co^3+^ into Co^2+^ on Electrochemically Reconstructed CoO_*x*_H_*y*_ Surface NF: nickel foam.

In 2023, Long’s group demonstrated a class of bifunctional electrocatalysts (Co_2_P_4_O_12_/NF) that can couple cathode HER (hydrogen evolution reaction) with anode BA-EODH (electrochemical oxidative dehydrogenation of benzylamine) to reduce power consumption significantly (Scheme 14). Due to the advantageous thermodynamic and kinetic properties of BA-EODH, this proposed configuration only requires a battery voltage as low as 1.47 V to provide a current density of 100 mA/cm^2^, saving up to 17% energy compared to traditional water splitting.^73^

This study achieved significant progress in selectivity, yield, and Faraday efficiency. However, the research mainly focuses on electrode materials; further development is needed on the substrate scopes.

#### 2.1.3 Application of MOF Electrodes in Amine Oxidation

Metal–organic frameworks (MOFs) have received widespread attention in recent decades due to their large surface area, variable porosity, and adaptability.^74^ Researchers have widely used them in electrodes and catalysts for electrochemical reactions.^75^

**Scheme 15:**
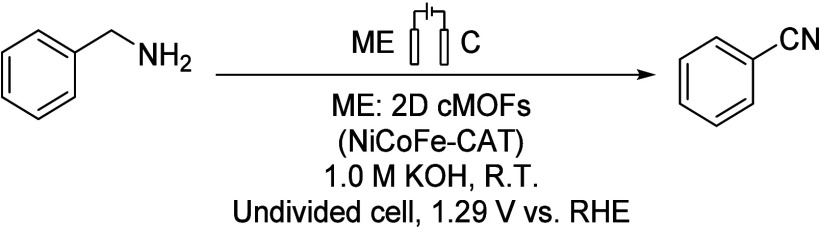
Application of NiCoFe-CAT MOFs in the Electrocatalytic Oxidation of Benzylamine to Benzonitrile CAT: hexahydroxytriphenylene linker. ME: metal electrode. RHE: reversible hydrogen electrode.

Many works have achieved the electrooxidation of benzylamine to benzonitrile on a series of multimetallic two-dimensional metal–organic frameworks (2D-cMOFs).^76,77^ The oxidation of benzylamine is carried out on a standard three-electrode system, which includes WEs such as Ni-CAT, NiCo-CAT, NiFe-CAT, and NiCoFe-CAT MOF nanowires, graphite as CE, and Hg/HgO as RE (Scheme 15). The three-metal skeleton NiCoFe-CAT exhibits the best performance, and due to its large active sites and durability, the NiCoFe-CAT provides good results under low potential. A bimetallic two-dimensional MOF catalyst was synthesized using an anodic electrochemical oxidation method, and benzonitrile was synthesized from benzylamine.

In 2022, Xu’s group reported on a membrane-free strategy achieved by oxidizing primary amines and coupling HER in a dual-electrode (t-Ni/Co MOF||Pt) electrolysis system. Various primary amines can be smoothly converted to the corresponding nitriles over the t-Ni/Co MOF anode in high yields and FEs. Due to the synergistic effect between Co and Ni, t-Ni/Co MOF can achieve benzylamine oxidation even at an ultralow potential of 1.30 V.^78^

**Scheme 16:**
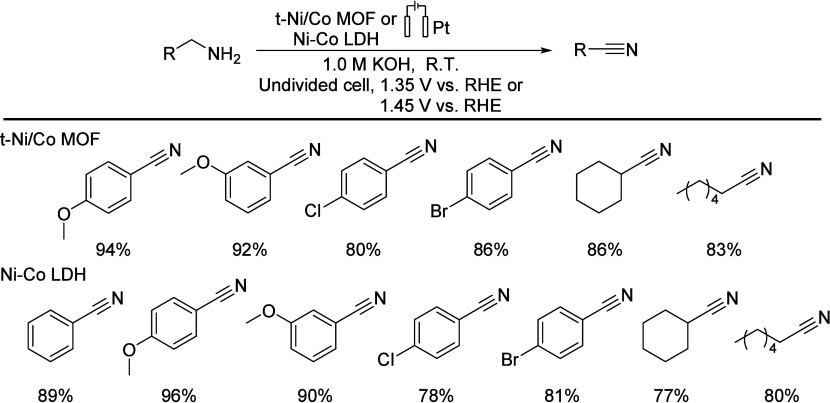
MOF and COF Electrochemical Oxidation of Amine Compounds to Nitriles LDH: layered double hydroxide. RHE: reversible hydrogen electrode.

The following year, the research group used a Co ZIF (zeolitic imidazolate framework) as a precursor to easily prepare a LDH (layered double hydroxide) at room temperature through simple immersion operations. The readily available LDH electrodes were successfully applied to organic electrooxidation reactions and individual hydrogen and oxygen evolution reactions. The obtained Ni–Co LDH electrode can provide high-density trivalent metal active sites and exhibit low charge transfer resistance to primary amine electrooxidation, thereby producing various nitriles in good yields (Scheme 16).^79^

The application of MOFs has improved the efficiency and selectivity of the electrosynthesis of nitrile compounds, and the yields have also been increased, making them suitable for various substrates. However, due to the complex preparation process of MOFs, their application as electrode materials is limited in organic electrosynthesis.

In 2020, Zhai’s group^4^ based on the surface-deficient Ni(OH)_2_ atomic layer (VR-Ni(OH)_2_, vacancy-rich Ni(OH)_2_) synthesized ultrathin Ni(OH)_2_ nanosheets via the in situ electrochemical conversion from Ni-MOFs nanosheets. Compared with VP-Ni(OH)_2_ (vacancy-poor Ni(OH)_2_), Ni(OH)_2_, which is rich in vacancies, effectively promoted the electrooxidation of amino C–N bonds to C≡N bonds.

**Scheme 17:**
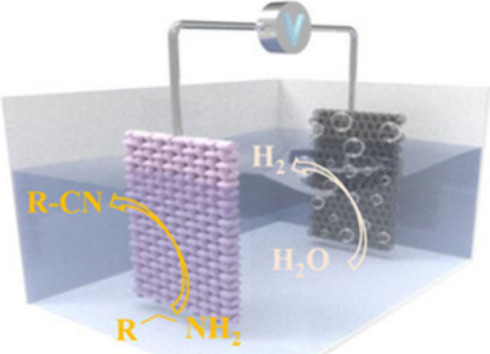
Schematic Presentation of the Homemade Propylamine Electrolyzer Using VR-Ni(OH)_2_ as the Anode and CoS_2_–MoS_2_ as the Cathode Reprinted with permission from ref (4). Copyright 2020 John Wiley and Sons.

In this research, VR-Ni(OH)_2_ was utilized as the anode and CoS_2_–MoS_2_ as the cathode with propylamine as the model substrate in an undivided cell (Scheme 17).^4^

**Scheme 18:**
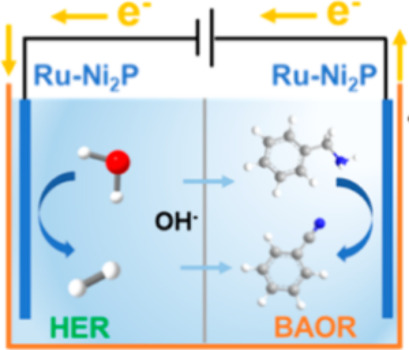
Scheme of the Two-Electrode Electrolyzer Using Ru–Ni_2_P/NF as Both Anode and Cathode HER: hydrogen evolution reaction. BAOR: benzylamine oxidation reaction. NF: nickel foam.

Transition metal compounds (TMCs) have been widely used in organic electrochemical synthesis research, especially nickel-based nanomaterials, including their phosphides, sulfides, and nitrides.^80−83^ However, due to the unoptimized chemical composition and electronic structure, the catalytic performance and dual functional performance of the catalyst are still unsatisfactory. Therefore, in 2024 Chen’s group reported that ruthenium can optimize the electronic structure of TMCs, and strengthen their electrocatalytic activities.^84^ They reported that a Ru-doped Ni_2_P nanobelt array assembled on nickel-foam (Ru–Ni_2_P/NF) showed excellent reactivity with ∼96.3% FE for producing benzonitrile from benzylamine (Scheme 18).

### 2.2 Electrochemical Synthesis of Imine Compounds

Imines serve as crucial building blocks, and the selective oxidation of amines to prepare imines constitutes a significant area of interest in organic synthesis. This topic has attracted considerable attention from researchers over the past few decades. However, the oxidation of amines often has drawbacks, such as the requirement for transition metal or even precious metal catalysts.^32^ Therefore, the electrochemical oxidation of amines to imines can be considered an environmentally friendly green method compared to classical routes.^67,79^

In 2005, Sasaki’s group used secondary amines to synthesize imines under alkaline conditions in an undivided cell at 15 °C, with platinum net as an anode and nickel coil as a cathode. According to their results, a catalytic amount of KI can cooperate with a strong base like NaOMe to achieve relatively high yields (62%–86%, Table 1).^85^

**Table 1:**
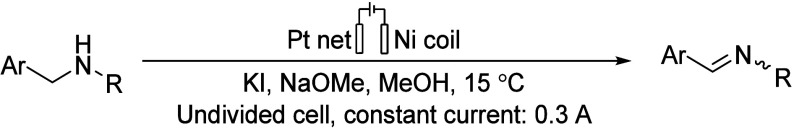

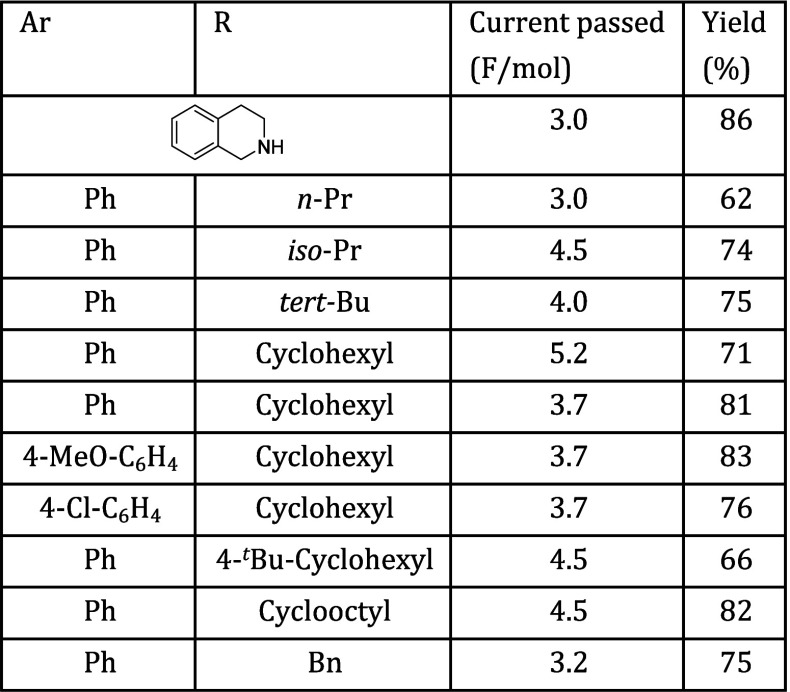
Electrooxidative Conversion of Benzylamines into the Corresponding Imines

**Scheme 19:**
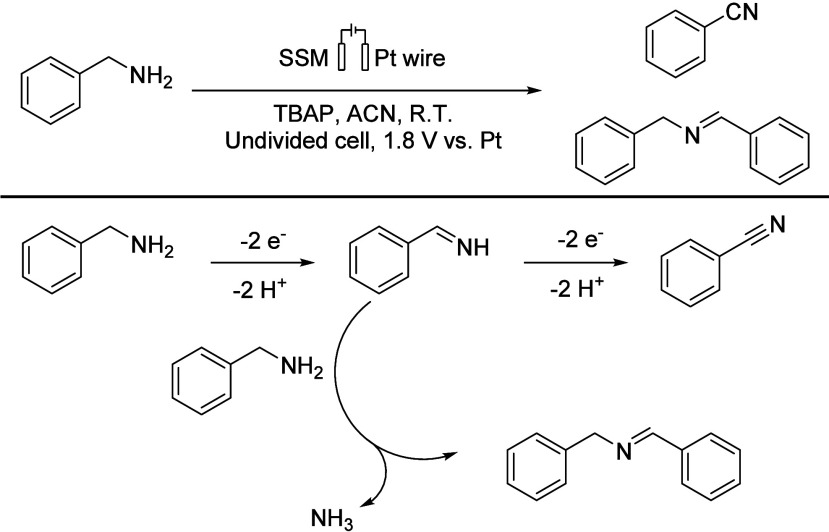
Proposed Reaction Mechanism for the Electrochemical Oxidation of Benzylamine SSM: stainless steel mesh. TBAP: tetrabutylammonium perchlorate.

Typically, noble metal-based materials such as gold, palladium, and platinum are considered the most effective catalysts for amine and alcohol oxidation.^86−89^ For example, Au–Pd alloy nanoparticles loaded on carbon nanotubes exhibit a 95% conversion rate for the oxidative self-coupling of benzylamine.^90^ However, due to the scarcity and high cost of these precious metals, they cannot be widely used. In electrochemistry, steel can be used as electrodes or catalytic carriers due to its high conductivity and good availability/accessibility. Kwon’s group (2022) used stainless steel as WE and Pt as CE without any added oxidant (Scheme 19). For benzylamine oxidation, stainless steel shows much higher reactivity compared to Pt.^18^

**Scheme 20:**
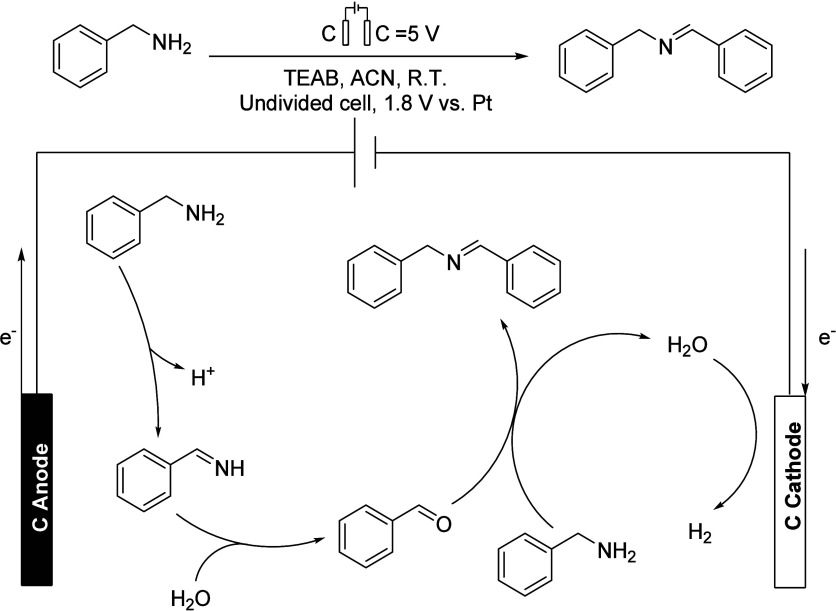
Synthesis of *N*-Benzyl-1-phenylmethanimine (top) and Its Proposed Mechanism in the Surface of Carbon Electrodes (bottom) TEAB: Et_4_NBr.

In 2022, Huang’s group subjected benzylamine to 10 h of self-oxidative coupling reaction under constant voltage (5 V) using tetraethylammonium bromide (TEAB) as an electrolyte. They applied an undivided cell equipped with a carbon anode and carbon cathode at room temperature, resulting in the formation of the target product imine with a yield of 96% (GC, Scheme 20).^32^

### 2.3 Electrocatalytic Methods for the Preparation of Nitrile Compounds from Amines

Several electrooxidation methods of amines to nitriles or imines have been developed successfully using nitroxyl radicals or halogen ions as mediators in the last decades.

#### 2.3.1 Application of TEMPO Derivatives As Mediators in Electrochemical Nitrile Synthesis

**Scheme 21:**
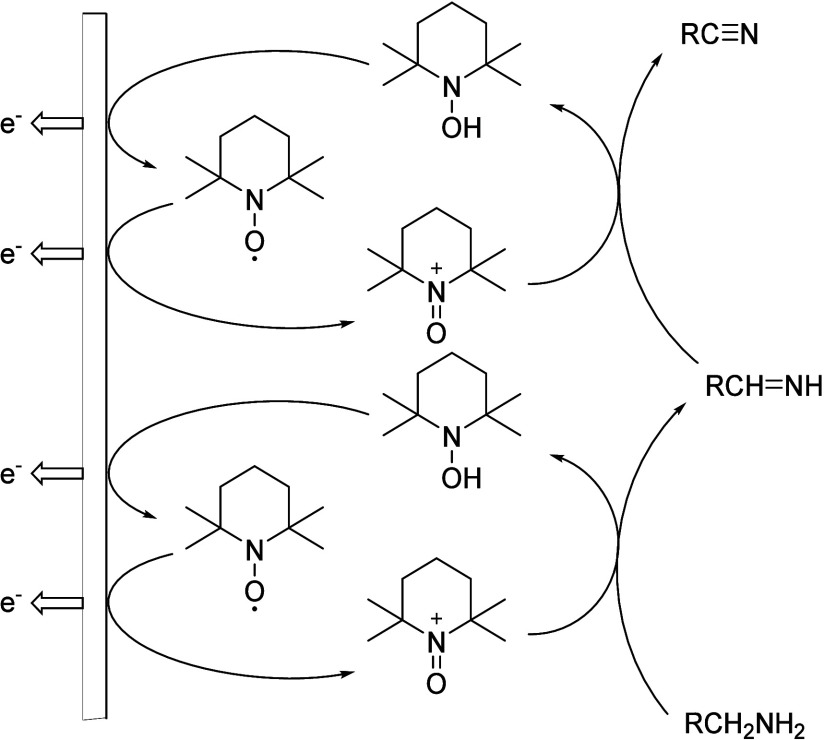
Proposed Mechanism of Electrocatalytic Oxidation of Amines to Nitriles Using TEMPO as an Additive

**Scheme 22:**
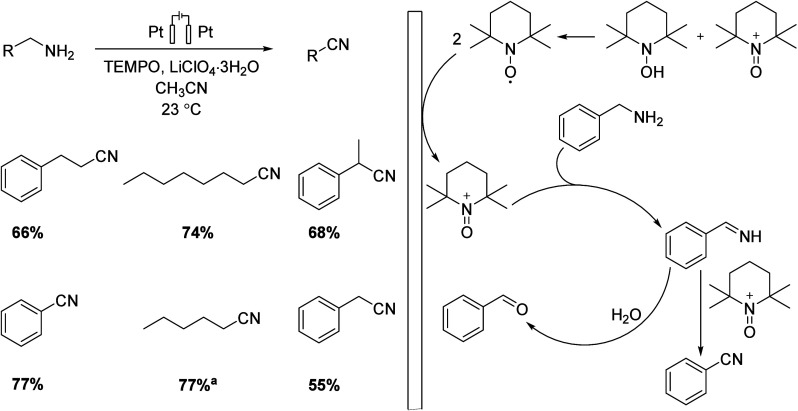
TEMPO Applied Effectively as a Mediator for Electrocatalytic Oxidation of Amine Compounds Isolated yields were determined by GLPC analysis.

TEMPO (2,2,6,6-tetramethylpiperidin-1-yloxyl) is an effective redox reagent that can be used for the oxidation of various organic compounds. In 1983, amines were successfully oxidized to nitriles by electrooxidation using a catalytic amount of TEMPO (Scheme 21). In the presence of water, unwanted carbonyl compounds were also formed (Scheme 22). In this work, Pt was applied to both WE and CE in an H-type cell, and LiClO_4_·3H_2_O in acetonitrile was used as an electrolyte. The isolated yields were high to moderate.^17^

Many works reported that electrodes such as graphite and platinum with thin polylayers can be used for electrocatalytic oxidation of amines.^91,92^

In 1998, Kashiwagi’s group reported a TEMPO-modified graphite electrode that can carry out the oxidation of amines to nitriles, using an H-type cell, selecting 2,6-lutidine and NaClO_4_ as cathodic electrolyte, Pt wire as CE, and Ag/AgCl as RE (Table 2).^93^

**Table 2:**
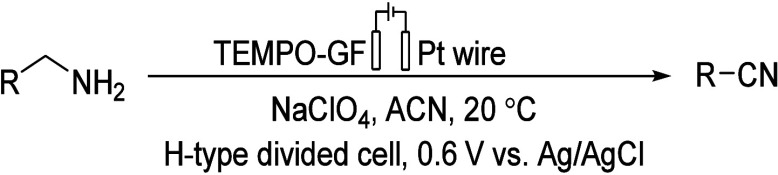
Electrocatalytic Oxidation of Amines on a TEMPO-Modified GF Electrode^a^

substrate	product	*Q* (C)	CE (%)	Con (%)	Sel (%)	TON
PhCH_2_NH_2_	PhCN	361	93.5	70.9	97.2	284
Ph(CH_2_)_2_NH_2_	PhCH_2_CN	374	94.2	74.5	97.8	298
*p*-MeO C_6_H_4_CH_2_NH_2_	*p*-MeO C_6_H_4_CN	362	92.6	68.7	95.3	275
CH_3_(CH_2_)_8_NH_2_	CH_3_(CH_2_)_7_CN	399	96.4	82.1	98.6	328

a GF: graphite felt. CE: Current efficiency. Con: conversion. Sel: Selectivity. TON: turnover number.

**Scheme 23:**
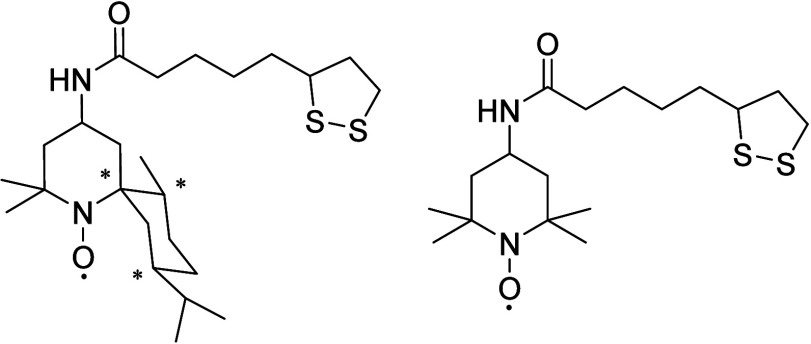
Structure of Achiral and Chiral TEMPO Derivatives Applied for the Gold Electrode Modification

In 1999, Kashiwagi’s group found enantioselective voltammetric behavior of chiral amines on a monolayer-modified gold electrode with a mixture of chiral and nonchiral nitro radical compounds and hexadecane thiol (Scheme 23, Table 3). In these experiments, Ag/AgCl was applied as reference (0.8 V).^92^

**Table 3:**
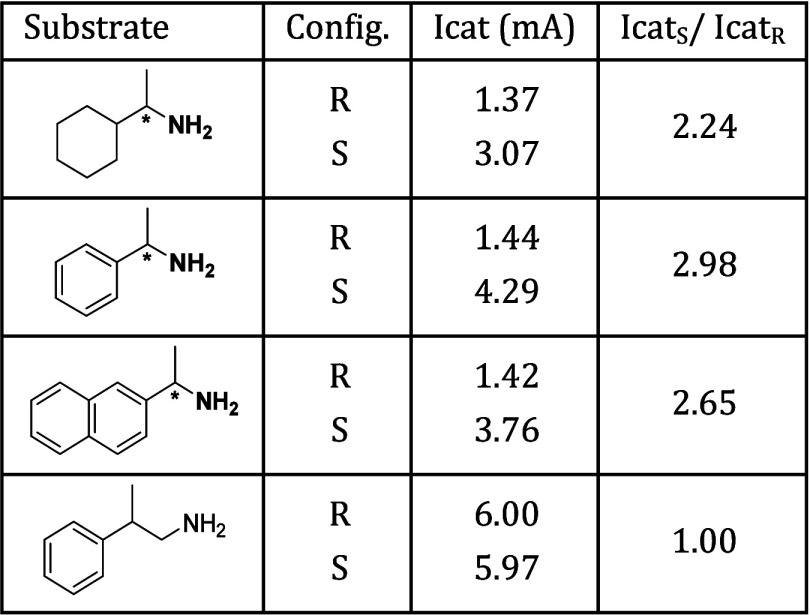
Enantioselective Voltammetric Behavior of Chiral Amines on a Monolayer-Modified Gold Electrode with a Mixture of Chiral and Non-chiral Nitro Radicals

The mechanism of oxidation in the presence of these additives is the same as the mechanism shown in Scheme 20, and the chiral induction of the ligand was happening by different steric interactions of the chiral ligand and the chiral substrates.

In 2013, the research group continued their research as they reported an effective method for immobilizing TEMPO catalysts on electrode surfaces through electrochemical copolymerization of 2,2′-dithiophene and precursors of TEMPO catalysts containing pyrrole side chains. It showed high reactivity toward the oxidation reactions of primary and secondary amines.^94^

#### 2.3.2 Nortropine *N*-Oxyl (NNO) as an Effective Mediator for Electrooxidation to Nitriles

**Scheme 24:**
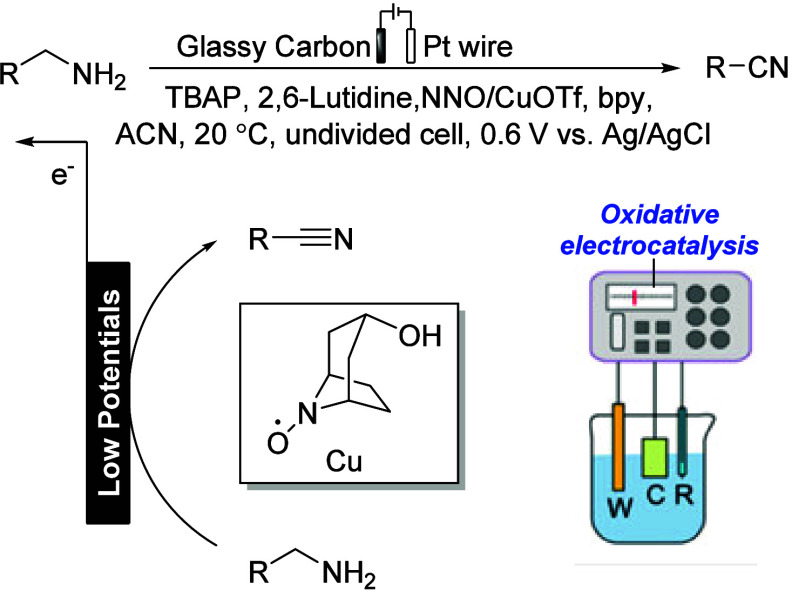
NNO/Copper Co-catalyst Applied for the Electrooxidation of Amines bpy: 2,2′-bipyridyl. NNO: nortropine *N*-oxyl. TBAP: tetrabutylammonium perchlorate. W, C, and R represent the working, counter, and reference electrodes, respectively. Reproduced in part with permission from ref (96). Copyright 2021 Pharmaceutical Society of Japan.

Electrochemical sensors, particularly biosensors based on enzyme reactions, offer high substrate specificity and temporal resolution due to the inherent characteristics of enzyme reactions. This enables highly reliable and rapid measurements without the need for preprocessing. However, the widespread utilization of these sensors is hindered by the poor long-term stability and high cost of enzymes.^95^ Kashiwagi has developed enzyme-free electrochemical sensing of alcohol and amine compounds using nitroxyl radical catalysts as organic catalysts (Scheme 24).^96^ Furthermore, after the successful tests, the NNO/copper catalysis can improve the oxidation current at a lower potential; the reactive state is better than that of TEMPO.

#### 2.3.3 Application of Halogen Mediators in Primary or Secondary Amine Electrooxidation

Indirect electrooxidation using a halogen mediator enables the oxidation of organic compounds using a catalytic amount of mediator. In 1984, Shono’s group reported the application of NaBr as a bromide ion source and a carbon rod and platinum as cathode and anode, respectively, in an electrochemical cell. The desired nitriles were prepared effectively from the primary amines. In the case of the reaction of secondary amines, the desired imines were detected in moderate to low yields (Table 4).^97^

**Table 4:**
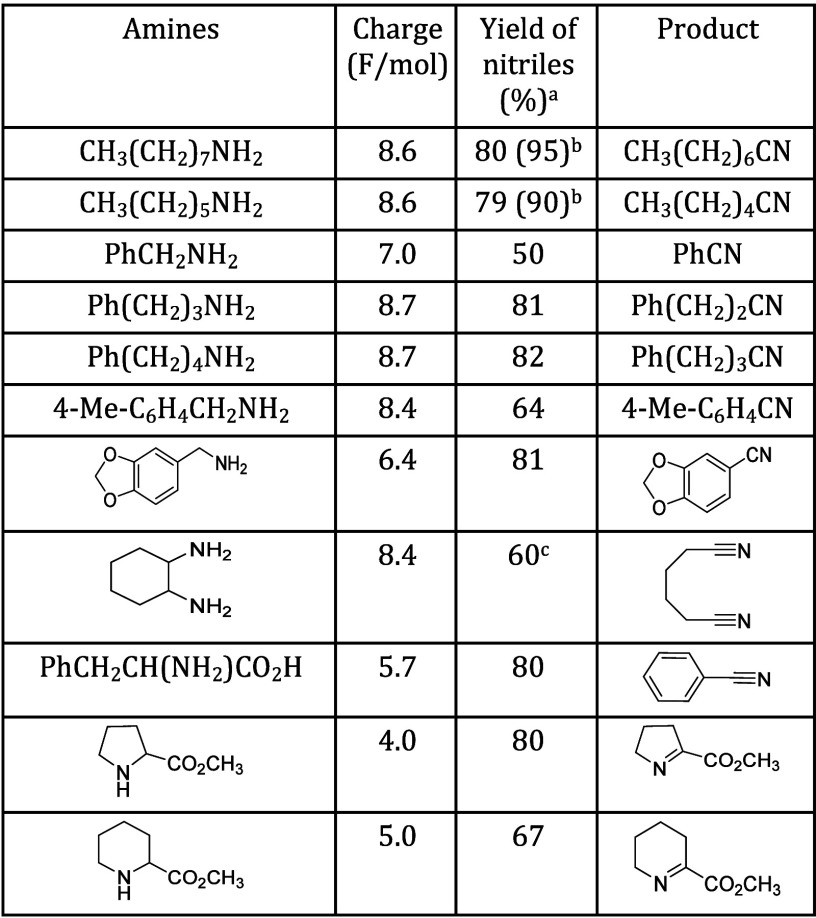
Electrooxidation of Amines Using Bromide Mediator^d^

a Isolated yields.

b Determined by gas–liquid chromatography.

c Adiponitrile. CP: Charge passed.

d MeOH, 30 mL; NaBr, 6 mmol; amine, 4 mmol.

In 2024, a method with modified bromine-mediated efficient electrochemical oxidation of amine to nitrile was published. This work utilized a highly efficient CoS_2_/CoS@graphite felt electrode. An impressive selectivity with remarkable Faradaic efficiency was achieved for both aliphatic and aromatic primary amines, highlighting its promising potential for practical applications.^98^

### 2.4 Electrocatalytic Transformation of Amines to Imines

#### 2.4.1 MNO as a Mediator for the Oxidative Synthesis of Imines

**Scheme 25:**
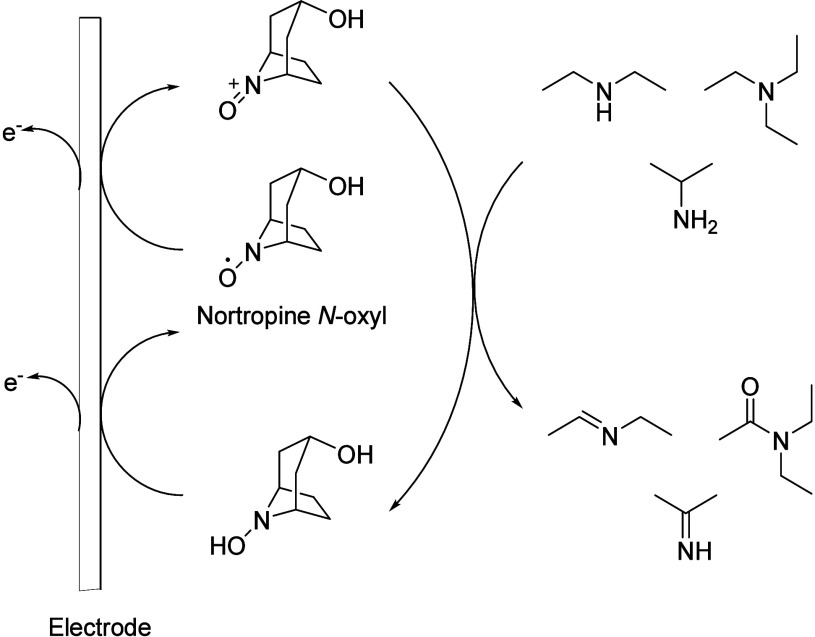
Nortropine *N*-Oxyl Mediated Oxidation with Primary, Secondary, and Tertiary Amines

Due to the lower reactivity of TEMPO compared to NNO, in 2018, Sato and colleges^99^ examined the electrooxidation ability of NNO in an electrochemical cell using glassy carbon as WE, Pt wire as CE, and Ag/AgCl (0.6 V) as RE. The experimental results indicated that NNO can electrochemically oxidize primary, secondary, and tertiary amines and isopropylamine under physiological conditions effectively (Scheme 25).^100^

#### 2.4.2 Triaryl Amines As Mediators in Electrocatalytic Imine Synthesis

Indirect anodic oxidation of amines to imines using aryl amine additives was successfully achieved. In these reactions, a triarylamino radical cation was formed, which proved to be a useful oxidizing agent. Various media have been tested in this research work including organic and inorganic media. Among the more successful organic media for oxidation are p-substituted triarylamines. Hence, in 1989, Pletcher and Zappi reported brominated triaryl amines as mediators to take part in the electrooxidation of amines to nitriles. In this electrochemical reaction, the WE was a vitreous carbon disc, and the CE was Pt gauze with Ag/AgNO_3_ as RE. This work shows that this mediator is a very effective additive; several benzylamines were transformed to the corresponding imines with high yields (Table 5).^101^

**Table 5:**
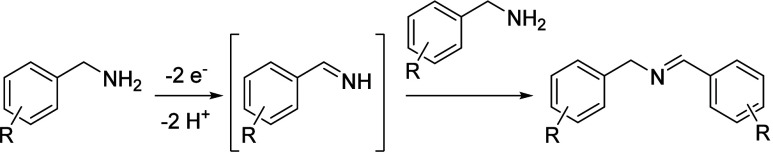
Products and Yields from the Electrolysis of Amine (0.1 mol dm^–3^) Using Tris(4-bromophenyl)amine (18 mmol dm^–3^) Containing 0.2 mol dm^–3^ NaClO_4_·H_2_O in Methanol/DCM (50/50 vol%) Using Undivided Cell, Vitreous Carbon Anode, and Pt Cathode

amine	amine consumption (%)	yield (%)
PhCH_2_NH_2_	95	imine (78)
4-Me-C_6_H_4_CH_2_NH_2_	90	imine (92)
4-MeO-C_6_H_4_CH_2_NH_2_	95	imine (86)
2,4-Cl_2_–C_6_H_3_CH_2_NH_2_	74	imine (65)
Ph_2_CHNH_2_	81	Ph_2_C=N–N=CPh_2_ (95)

## 3 Conclusion

This review aims to provide a foundational understanding that can promote further research endeavors in the electrocatalytic oxidation of amines. It highlights a variety of electrodes and experimental conditions that have been investigated over the past several decades. While these electrodes offer advantages such as high conversion rates, high Faradaic efficiency, and high yields, the scope of substrates investigated is limited and their production processes are complex. Traditional electrodes such as graphite, stainless steel, and lead are inexpensive, have a wide range of applications, and in some cases provide high yields. Applying mediators such as bromine, N-oxides, and N-oxide-modified electrodes in electrocatalytic methods developed in recent years produced enhanced reactivity. Based on the results achieved in the field of electrochemical transformation of amines, electrochemistry seems to be a versatile and sustainable method for the preparation of aromatic and aliphatic nitriles and imines; however, further research and development are needed in this aspect. The substrate scope is limited in the case of the electrochemical oxidation of amines. In the presence of aryl, alkyl, or alkoxy groups or bromine and chlorine moieties, the desired products were formed, but other functional group tolerances have not been published yet.

## Data Availability

The data underlying this study are available in the published article.
